# Supplementary material for: Circuit and synaptic organization of forebrain-to-midbrain pathways that promote and suppress vocalization
Source: eLife. 2020 Dec 29;9:e63493. doi: 10.7554/eLife.63493 (PMC7793624; doi:10.7554/eLife.63493)
Supplement: Figure 1—source data 1. [file elife-63493-fig1-data1.docx]

| **Cortical regions** | VGAT+ PAG | PAG-USV |
| --- | --- | --- |
| Prelimbic/infralimbic | + | + |
| Insular | + | - |
| M1/M2 | + | + |
| Cingulate | + | + |
| S1/S2 | + | + |
| Auditory | + | - |
| **Subcortical** | VGAT+ PAG | PAG-USV |
| Ventral pallidum | + | + |
| Lateral septum | + | + |
| BNST | + | + |
| Habenula | + | + |
| Zona incerta | + | + |
| **Hypothalamus** | VGAT+ PAG | PAG-USV |
| Preoptic | + | + |
| Anterior hypothalamus | + | + |
| Lateral hypothalamus | + | + |
| PVH | + | + |
| VMH | + | + |
| Posterior hypothalamus | + | + |
| Premammillary/mammillary | + | + |
| **Amygdala** | VGAT+ PAG | PAG-USV |
| Extended amygdala | + | + |
| Central amygdala | + | + |
| **Thalamus** | VGAT+ PAG | PAG-USV |
| PVT | + | + |
