# Supplementary material for: Circuit and synaptic organization of forebrain-to-midbrain pathways that promote and suppress vocalization
Source: eLife. 2020 Dec 29;9:e63493. doi: 10.7554/eLife.63493 (PMC7793624; doi:10.7554/eLife.63493)
Supplement: Transparent reporting form [file elife-63493-transrepform.docx]

***eLife’s* transparent reporting form**

We encourage authors to provide detailed information *within their submission* to facilitate the interpretation and replication of experiments. Authors can upload supporting documentation to indicate the use of appropriate reporting guidelines for health-related research (see [EQUATOR Network](http://www.equator-network.org/%20)), life science research (see the [BioSharing Information Resource](https://biosharing.org/)), or the [ARRIVE guidelines](http://www.plosbiology.org/article/info:doi/10.1371/journal.pbio.1000412) for reporting work involving animal research. Where applicable, authors should refer to any relevant reporting standards documents in this form.

If you have any questions, please consult our Journal Policies and/or contact us: [editorial@elifesciences.org](mailto:editorial@elifesciences.org).

**Sample-size estimation**

- You should state whether an appropriate sample size was computed when the study was being designed
- You should state the statistical method of sample size computation and any required assumptions
- If no explicit power analysis was used, you should describe how you decided what sample (replicate) size (number) to use

Please outline where this information can be found within the submission (e.g., sections or figure legends), or explain why this information doesn’t apply to your submission:

No statistical analyses were used to pre-determine sample size. This information can be found in “Quantification and Statistical Analyses” within our Methods section. Throughout the manuscript, we include sample sizes of at least N=4 per group, with the exception of the bilateral axon terminal optogenetic activation experiments (which were extremely technically challenging and resulted in a higher mortality rate than for any of our other optogenetic experiments). For in situ hybridization, we used N=2 mice per experiment, which is standard in the field. For whole-cell recordings, we obtained a sample size of at least N=10 cells per experiment. The sample sizes used in each experiment are reported in the Results text, as well as within the relevant figures and figure legends.

**Replicates**

- You should report how often each experiment was performed
- You should include a definition of biological versus technical replication
- The data obtained should be provided and sufficient information should be provided to indicate the number of independent biological and/or technical replicates
- If you encountered any outliers, you should describe how these were handled
- Criteria for exclusion/inclusion of data should be clearly stated
- High-throughput sequence data should be uploaded before submission, with a private link for reviewers provided (these are available from both GEO and ArrayExpress)

Please outline where this information can be found within the submission (e.g., sections or figure legends), or explain why this information doesn’t apply to your submission:

We employed biological replication in this manuscript (i.e., different animals or cells were tested using the same procedure, to determine the across-sample variability in our results). We did not employ technical replication (i.e., the same animal or cell tested multiple times to determine within-sample variability). The numbers of animals and cells tested are reported within the Results text for each experiment, as well as within the relevant figures and figure legends. Animals were only excluded from analysis in cases in which viral injections were mis-targeted, or in which viral expression was poor or absent. This information can be found in “Quantification and Statistical Analyses” within our Methods section.

**Statistical reporting**

- Statistical analysis methods should be described and justified
- Raw data should be presented in figures whenever informative to do so (typically when N per group is less than 10)
- For each experiment, you should identify the statistical tests used, exact values of N, definitions of center, methods of multiple test correction, and dispersion and precision measures (e.g., mean, median, SD, SEM, confidence intervals; and, for the major substantive results, a measure of effect size (e.g., Pearson's r, Cohen's d)
- Report exact p-values wherever possible alongside the summary statistics and 95% confidence intervals. These should be reported for all key questions and not only when the p-value is less than 0.05.

Please outline where this information can be found within the submission (e.g., sections or figure legends), or explain why this information doesn’t apply to your submission:

Parametric, two-sided comparisons were used unless otherwise noted (alpha=0.05). This information can be found in “Quantification and Statistical Analyses” within our Methods section. The statistical test employed for each analysis, the exact sample sizes employed, and methods of multiple test correction (where applicable) are reported parenthetically within the relevant portion of the Results section for each experiment.

(For large datasets, or papers with a very large number of statistical tests, you may upload a single table file with tests, Ns, etc., with reference to sections in the manuscript.)

**Group allocation**

- Indicate how samples were allocated into experimental groups (in the case of clinical studies, please specify allocation to treatment method); if randomization was used, please also state if restricted randomization was applied
- Indicate if masking was used during group allocation, data collection and/or data analysis

Please outline where this information can be found within the submission (e.g., sections or figure legends), or explain why this information doesn’t apply to your submission:

Mice were selected at random for inclusion into either experimental or control groups for optogenetic experiments. This information can be found in “Quantification and Statistical Analyses” within our Methods section. Masking was not used during data collection or analysis, but automated Matlab codes were applied to data from each animal in an identical manner to generate analyzed data (for example, to detect USVs, to measure mouse movement speed, to measure the amplitude of optogenetically-elicited post-synaptic currents, etc.).

**Additional data files (“source data”)**

- We encourage you to upload relevant additional data files, such as numerical data that are represented as a graph in a figure, or as a summary table
- Where provided, these should be in the most useful format, and they can be uploaded as “Source data” files linked to a main figure or table
- Include model definition files including the full list of parameters used
- Include code used for data analysis (e.g., R, MatLab)
- Avoid stating that data files are “available upon request”

Please indicate the figures or tables for which source data files have been provided:

We are not including additional source data at this time.
